# Multi-source data recognition and fusion algorithm based on a two-layer genetic algorithm–back propagation model

**DOI:** 10.3389/fdata.2024.1520605

**Published:** 2025-01-13

**Authors:** Zhuang Xiong, Jun Ma, Bohang Chen, Haiming Lan, Yong Niu

**Affiliations:** ^1^The College of Computer, Qinghai Normal University, Xining, China; ^2^Department of Mechanical and Electrical Engineering, College of Xining Urban Vocational and Technical, Xining, China

**Keywords:** multi-source data fusion, BP neural network, legacy algorithm, genetic algorithm–optimized back propagation network, multi-sensor fault recognition

## Abstract

Traditional rainfall data collection mainly relies on rain buckets and meteorological data. It rarely considers the impact of sensor faults on measurement accuracy. To solve this problem, a two-layer genetic algorithm–backpropagation (GA-BP) model is proposed. The algorithm focuses on multi-source data identification and fusion. Rainfall data from a sensor array are first used. The GA optimizes the weights and thresholds of the BP neural network. It determines the optimal population and minimizes fitness values. This process builds a GA-BP model for recognizing sensor faults. A second GA-BP network is then created based on fault data. This model achieves data fusion output. The two-layer GA-BP algorithm is compared with a single BP neural network and actual expected values to test its performance. The results show that the two-layer GA-BP algorithm reduces data fusion runtime by 2.37 s compared to the single-layer BP model. For faults such as lost signals, high-value bias, and low-value bias, recognition accuracies improve by 26.09%, 18.18%, and 7.15%, respectively. The mean squared error is 3.49 mm lower than that of the single-layer BP model. The fusion output waveform is also smoother with less fluctuation. These results confirm that the two-layer GA-BP model improves system robustness and generalization.

## 1 Introduction

Single-source signal processing or low-level multi-source data processing is a low-level imitation of external biological information processing. Multi-source data fusion (Jiao et al., 2023) makes full use of multi-sensor arrays to collect resource data, packages them into a single dataset, and then uses different algorithms to extract the required quantity of information. However, presently, data fusion faces by many urgent challenges, such as data defects, abnormal data, and data correlation. Therefore, research regarding multi-source data fusion is of great significance.

Regarding multi-source data recognition, classification, and prediction for data fusion (Chen et al., 2022; Jin et al., 2021), two key issues need to be addressed. First, we must solve the issue of multi-sensor fault recognition and classification at the data collection source. Second, to achieve multi-source data fusion output, we must construct an appropriate data model based on the characteristics of the fault data.

To address the issue of multi-sensor fault recognition, Wang et al. (2023) proposed a fault diagnosis method based on multi-sensor fusion and efficient channel attention for a convolutional neural network (ECA-CNN) is proposed. The results show that this method has strong generalization and high computational efficiency. Xu et al. (2022) researched a general method for fault diagnosis of complex systems using time series features and transfer entropy and then generalized its usage. Fu et al. (2022) found that gearbox fault diagnosis based on the multi-sensor genetic algorithm–backpropagation (GA-BP) algorithm is investigated, proposing a decision-level fault recognition method that integrates Dempster–Shafer (DS) evidence theory with the GA-BP algorithm, thereby significantly improving recognition accuracy. However, no feasible solutions were proposed for soft faults or data defects. For fault recognition, many studies have proposed a fault diagnosis solution based on variational mode decomposition (VMD), where source data are decomposed into modes. Methods such as Fourier and Hilbert transforms are used for time- and frequency-domain analysis to identify faulty nodes. As the VMD algorithm has optimization issues regarding the number of modes, many studies have focused on optimizing VMD for fault recognition (Zheng F. et al., 2023; Zheng Y. et al., 2023; Yu et al., 2023; Zhu et al., 2023; Yu et al., 2024; Huang, 2023), and great achievements have been made.

However, beyond multi-sensor fault recognition, further research is needed to troubleshoot faults and achieve multi-source data fusion output. This study introduces the BP neural network algorithm, using a GA to update the thresholds and weights of the BP neural network. A two-layer GA-BP network is constructed to achieve multi-sensor fault recognition and multi-source data fusion output. This model has the following key features:

- Through the research of this article, the accuracy of rainfall monitoring is improved, and data support is provided for the occasions and equipment with high rainfall accuracy, such as astronomical observation equipment.
- By constructing a two-layer GA-BP algorithm model, a complete fault recognition and data fusion system was designed, increasing the generalization ability of system.
- The GA-BP algorithm model improves the identification accuracy and running time compared to the single-layer BP neural network model. The fusion output of the two-layer GA-BP algorithm model has a “smooth” and stable output waveform, with small fluctuations and significantly reduced mean square error, thus improving the robustness of the system.

The structure of this article is as follows: This paper is divided into six sections. Section 2 briefly discusses research regarding multi-source data fusion and multi-sensor fault recognition and outlines the research ideas put forth in this paper. Section 3 describes the system model proposed in this paper. Section 4 describes the GA-BP-based multi-source data recognition and prediction model. Section 5 describes the experimental simulations and comparative analyses conducted in this study. Section 6 summarizes and concludes the paper.

## 2 Related research

Currently, multi-source data research still faces several challenges, caused mainly by defects in sensor technology, inaccurate fault recognition, and significant errors in data fusion output. Researchers primarily use methods such as BP neural network algorithms, GA-optimized BP neural network models, VMD algorithms, Fourier transform, and wavelet analysis for fault diagnosis and analysis (Sun et al., 2023). There are large amount of research existing on fault diagnosis and multi-source data fusion in the form of literature and algorithmic models, but there is very little research on integrating multi-sensor fault recognition with data fusion output.

### 2.1 Research regarding multi-sensor fault recognition and multi-source data prediction fusion

A BP neural network model considers an unknown system to be a “black box.” Complex, non-linear systems that are difficult to model mathematically are expressed through a specific network and then finally simulated and output through the trained BP neural network. However, this method suffers from the issue of local maximum and minimum values due to inappropriately selected parameters and therefore does not lead to a globally optimal solution. In addition, the network depends on the availability of large volume of training data and so is prone to issues such as insufficient training capacity and inaccurate prediction. To address these issues, many researchers have begun to optimize the BP neural network parameters using a GA. Specifically, the GA-BP model uses a GA to optimize the weights and thresholds of the BP neural network, generating an optimal population. This optimal population is used to build a neural network and to calculate the optimal fitness value output (Yu et al., 2022; Zheng et al., 2022). Wang et al. (2024) found that a GA was used to optimize the number of modes (k) and the penalty factor (α) in the VMD algorithm, decomposing data into k modal components and residuals. The experimental results demonstrate that this method has potential for application in sensor fault recognition and location. In Yang et al. (2024), aiming at the problems that abnormal values and uneven noise distribution often occur in power load data, Pinball-Huber is adopted as the robust loss function, and a prediction model based on the improved extreme learning machine (ELM) is proposed. The genetic algorithm is combined with the fast non-dominated sorting technique to conduct multi-objective optimization for the proposed method. This method effectively reduces the training error and the model structure.

As a part of further research, Li et al. (2022) used BP, GA-BP, and PSO-BP (particle swarm optimization–backpropagation) neural network algorithms to construct a short-term photovoltaic power generation model. Simulation results demonstrated that the GA-BP and PSO-BP networks achieved high predictive accuracy, indicating that the GA and PSO-optimized models effectively reduced prediction errors when compared with the original BP model. Liu et al. (2022) found that a GA-optimized BP neural network regression model is proposed for predicting high-slope soil moisture. The BP neural network regression model and the GA-BP neural network regression model were used for soil moisture prediction with and without lags. The results showed that both prediction methods exhibited a more significant improvement in prediction accuracy when considering lags compared with those without lags, with the GA-BP neural network regression model outperforming the BP neural network regression model in terms of accuracy. Tan et al. (2023) proposed, regarding the algorithm optimization problem, based on the firefly algorithm, a learning algorithm based on the adaptive logarithmic spiral–Lévy flight firefly algorithm (QLADIFA) is proposed. Experiments show that the proposed algorithm effectively overcomes the limitations of the firefly algorithm and effectively improves the performance of algorithm.

### 2.2 Research methods

To address the issues of fault recognition and data fusion, this study proposes a multi-source data recognition, classification, and prediction fusion algorithm based on a two-layer GA-BP model. The multi-source data are collected by multi-sensor arrays, and the BP and GA-BP neural network algorithms are used to locate faulty nodes and recognize normal, lost, and abnormal signals. The results of the two algorithms are compared and analyzed in terms of simulation time, recognition accuracy, and mean squared error to further validate that the GA-BP model outperforms the single BP neural network in fault recognition (Gong et al., 2022). In addition, hidden layer nodes and a mean squared error mathematical model are established in MATLAB, and iteratively, the optimal local solution of the hidden layers is realized. The recognized faulty nodes serve as inputs to the second-layer GA-BP model, which is compiled and debugged by specified sub-functions to obtain the optimal population and best fitness value, thus ultimately achieving multi-source data fusion output.

## 3 System model

**Figure 1:**
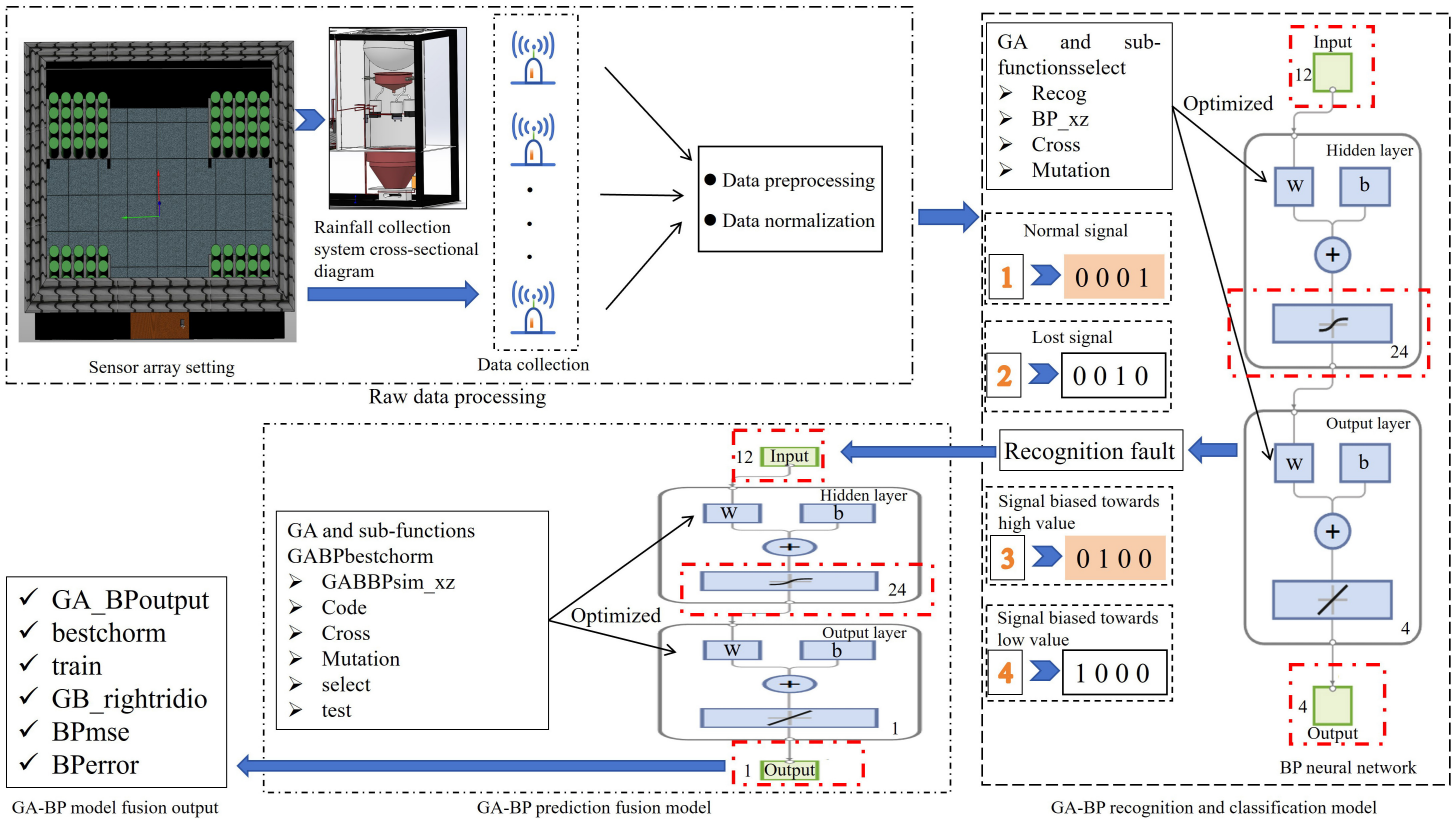
Overall design structure diagram of GA-BP.

The proposed method for multi-source data recognition, classification, and prediction fusion based on a two-layer GA-BP model consists of three modules: raw data processing, a GA-BP recognition and classification model, and a GA-BP prediction fusion model, as shown in Figure 1. The raw data processing module consists of three parts: 80-sensor nodes that collect rainfall data over 12 time intervals during a single day, a data preprocessing function, and a data normalization function. The GA-BP recognition and classification module comprises mainly a BP neural network (with 13 input nodes, 21 hidden layer nodes, and four output nodes), a GA, and sub-functions (select, Recog, BP_xz, cross, and mutation). The GA-BP prediction fusion model is primarily composed of a BP neural network (with 12 input nodes, nine hidden layer nodes, and one output node), a GA, and sub-functions (select, Recog, BP_xz, cross, and mutation).

## 4 GA-BP-based multi-source data recognition and prediction model

### 4.1 BP neural network

A BP neural network realizes the mapping from n-dimensional input to m-dimensional output. The signal passes from the input layer to the hidden layer and then to the output layer, where it is comparatively analyzed with the expected output. The error is backpropagated, and the weights and thresholds of the network are updated based on the prediction error. After multiple iterations, the predicted output gradually approaches the expected output.

The BP neural network undergoes mathematical modeling using a linear transfer function, without bias values, and containing one hidden layer. The specific expression is as follows (Bai et al., 2021):


(1) $$\begin{array}{l} y_{k}=\overset{N_{2}}{\underset{j=1}{\sum }}w_{kj}^{2}f\left(\overset{N_{1}}{\underset{i=1}{\sum }}w_{ji}^{1}x_{i}+b_{i}\right) \end{array}$$

where *y*_*k*_ is the k-th output; $w_{kj}^{2}$ is the weight of neuron j in layer 2 (the hidden layer) to neuron k in the output layer; *f* is the transfer function of the neuron in the hidden layer; $w_{\text{ji}}^{1}$ is the weight of neuron i in layer 1 (the input layer) to neuron j in the hidden layer; and *b*_*j*_ is the bias value of neuron j in the hidden layer. When the number of hidden layers is appropriately set, the BP neural network can accurately approximate any complex non-linear system function.

The specific algorithmic 1 steps are as follows:

**Algorithm 1:**
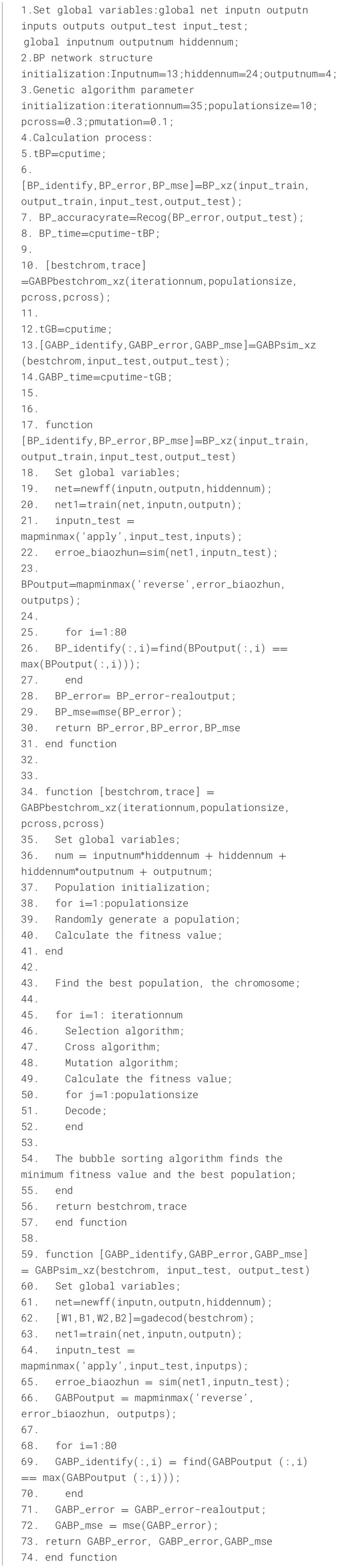
GA-BP multi-source data identification and fusion.

**Step 1**: Network initialization. Based on the system input and output, determine the number of input layer nodes m, the number of hidden layer nodes *l*, the number of output layer nodes n, and the weights between the layers *w*_*ij*_ (weight between the input layer and the hidden layer), *w*_*j*k_ (weight between the input layer and the hidden layer), the threshold of the hidden layer a, and the threshold of the output layer b.

**Step 2**: Hidden layer output. The specific modeling expression is shown in Equation 2, where *f* is the hidden layer excitation function.


(2) $$\begin{array}{l} H_{j}=f\left(\overset{n}{\underset{i=1}{\sum }}w_{ij}x_{i}-a_{j}\right)\;j=1,2,3\ldots \ldots ,1 \end{array}$$

**Step 3**: Mathematical modeling of the output layer. The hidden layer output function *H* is used in combination with the associated weights and thresholds to compute the predicted output *O*.


(3) $$\begin{array}{l} O_{k}=\overset{l}{\underset{j=1}{\sum }}H_{j}w_{jk}-b_{k}\;k=1,2,3\ldots \ldots ,m \end{array}$$

**Step 4**: Error calculation (Zheng F. et al., 2023; Zheng Y. et al., 2023). The prediction error *e* is calculated by taking the difference between the predicted output *O* and the expected output *Y* of the neural network.


(4) $$\begin{array}{l} e_{k}=Y_{k}-O_{k}\;k=1,2,3\ldots \ldots ,m \end{array}$$

**Step 5**: The weights and thresholds of the network nodes are updated based on the prediction error *e*.


(5) $$\begin{array}{l} \;w_{ij}=w_{ij}+\eta H_{j}\left(1-H_{j}\right)x\left(i\right)\overset{m}{\underset{k=1}{\sum }}w_{jk}e_{k}\\ i=1,2,3\ldots \ldots ,n;j=1,2,3\ldots \ldots ,l \end{array}$$

(6) $$\begin{array}{r} w_{jk}=w_{jk}+\eta H_{j}e_{k}\\ \;j=1,2,3\ldots \ldots ,l;k=1,2,3\ldots \ldots ,m \end{array}$$

(7) $$\begin{array}{r} a_{j}=a_{j}+\eta H_{j}\left(1-H_{j}\right)\overset{m}{\underset{k=1}{\sum }}w_{jk}e_{k}\\ \;j=1,2,3\ldots \ldots ,l \end{array}$$

(8) $$\begin{array}{l} b_{k}=b_{k}+e_{k}\;k=1,2,3\ldots \ldots ,m \end{array}$$

**Step 6**: Based on the number of iterations and the critical value of the prediction error, it is determined whether or not to stop the iterations. If not, return to step 2 and proceed to the next iteration.

### 4.2 Genetic algorithm (GA)

A GA simulates the process of natural selection, reproduction, and mutation through the iterative simulation of each generation of different random changes, so as to generate a set of candidate populations. Individuals are screened based on the selected fitness function and through genetic selection, crossover, and mutation. The higher the fitness value, the closer the population is to the optimal local solution. Better fitness values are retained, and so the new generation of candidate populations has better fitness values and populations than the previous. The population iterates until convergence criteria are met.

The GA consists of four parts: encoding to generate the initial population, fitness function, genetic operators (selection, crossover, and mutation), and control parameters, among which the three basic genetic operators are the most important.

#### 4.2.1 Selection operator

The selection operation is the process of selecting individuals from the parent population to pass onto the next generation. A higher fitness value indicates a greater probability of the individual being passed onto the next generation. The specific fitness value calculation is shown in Equation 9. The selection operation is conducted using the roulette wheel method, i.e., a selection strategy based on the fitness ratio. The selection probability *p*_*i*_ for an individual *i* is calculated using Equations 10, 11:


(9) $$\begin{array}{l} F=k\left(\overset{n}{\underset{i=1}{\sum }}abs\left(y_{i}-o_{i}\right)\right) \end{array}$$

(10) $$\begin{array}{l} f_{i}=k/F_{i} \end{array}$$

(11) $$\begin{array}{l} p_{i}=\frac{f_{i}}{\overset{N}{\underset{j=1}{\sum }}f_{j}} \end{array}$$

#### 4.2.2 Crossover operator

**Figure 2:**
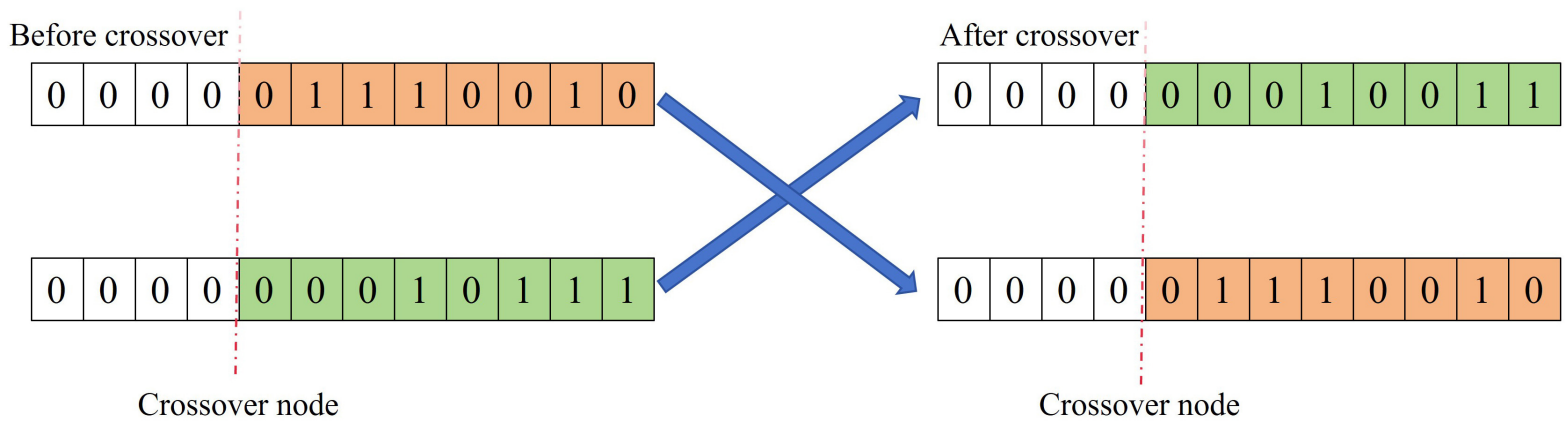
Schematic diagram of the crossover process of the genetic algorithm.

Two paired chromosomes exchange part of their genes with one another based on the crossover probability *P*_*c*_, thereby forming two new individuals. The detailed crossover process is shown in Figure 2. Suppose that the crossover of the k-th chromosome *a*_*k*_ and the *l*-th chromosome *a*_*l*_ at the *j*-th position is mathematically modeled as Equation 12, where b is a random number in the range [0, 1].


(12) $$\{\begin{array}{l} a_{kj}=a_{kj}(1-b)+a_{ij}b\\ a_{lj}=a_{lj}(1-b)+a_{kj}b \end{array}$$

#### 4.2.3 Mutation operator

**Figure 3:**
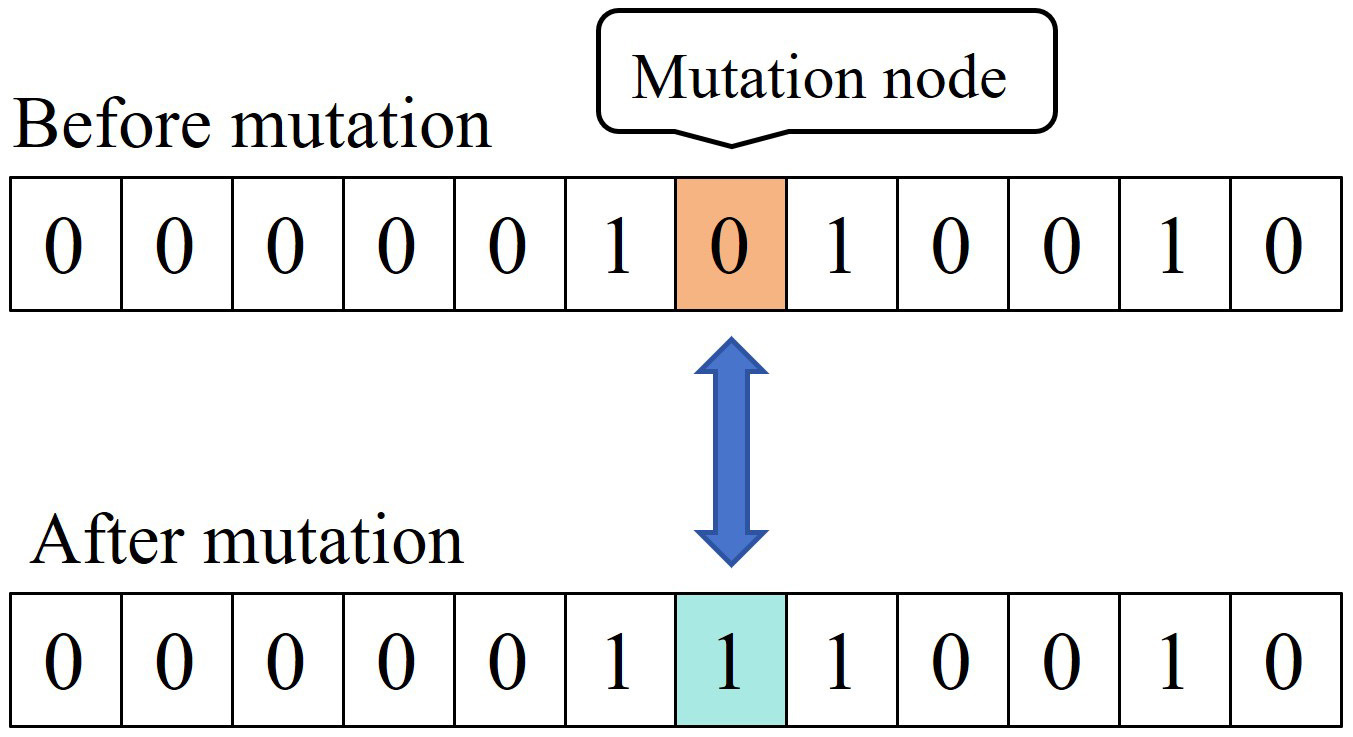
Schematic diagram of the mutation process of the genetic algorithm.

Based on the mutation probability *p*_*m*_, certain gene values in the individual coding string are replaced by other gene values, thereby creating a new individual. The detailed mutation process is shown in Figure 3 (Misevičius and Verene, 2021).

### 4.3 GA-BP-based multi-source data processing model

**Figure 4:**
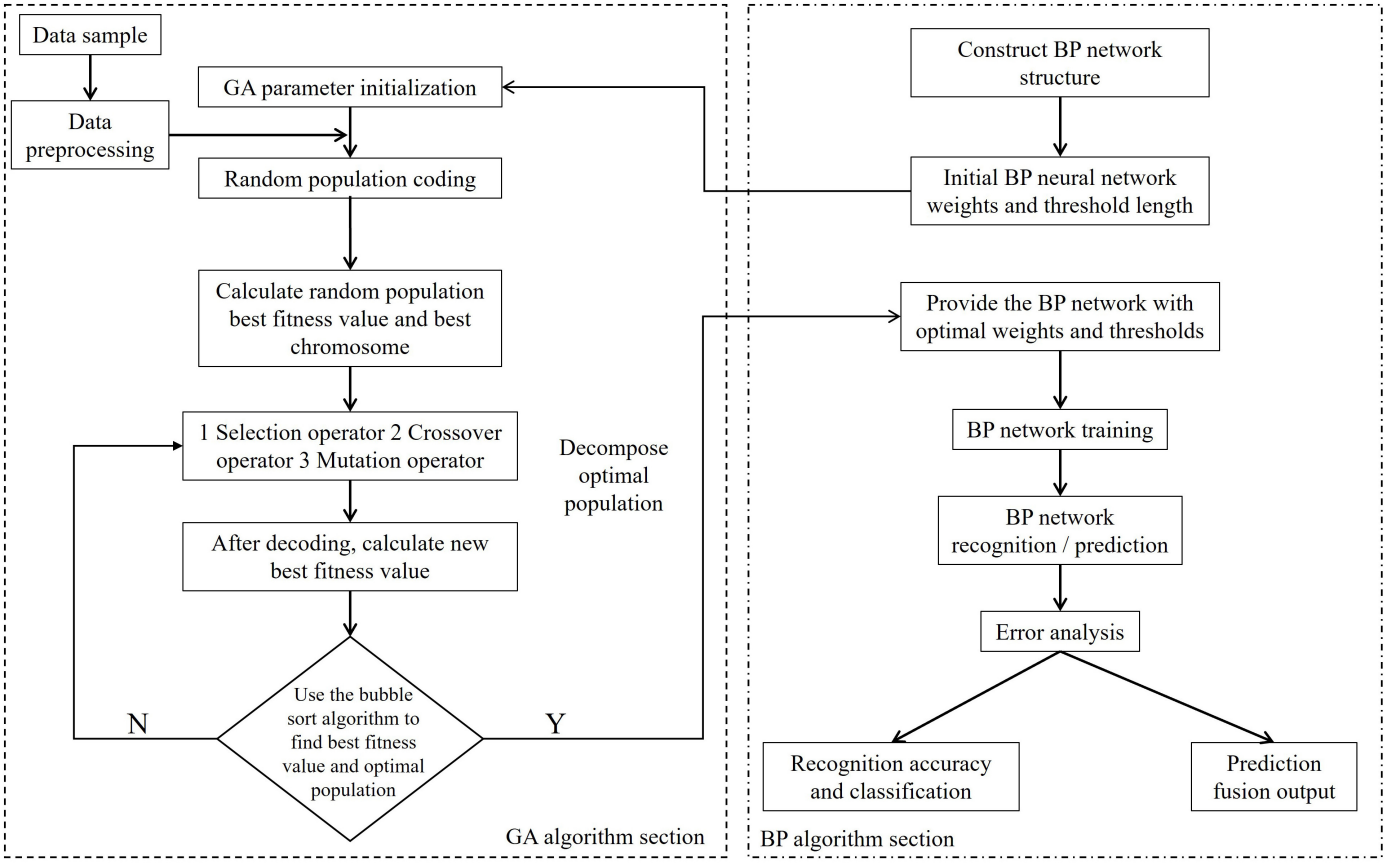
Flowchart of the GA-BP process.

The GA-BP-based multi-source data processing model combines the BP neural network model and the GA model (Jiang et al., 2024; Liu et al., 2023; Zhang et al., 2024; Wang et al., 2022). The detailed algorithm flowchart is shown in Figure 4. The BP neural network model recognizes and classifies sensor faults, precisely locates faulty nodes and fault types, and then performs data fitting and fusion output. Of the characteristic rainfall signals collected by the sensor arrays, 60 normal signal populations, 60 lost signal populations, 60 signal populations biased toward high values, and 60 signal populations biased toward low values are selected. Each population is further decomposed into 14 characteristic signals, of which 12 characteristic signals, one sequence type signal, and one total rainfall characteristic signal are collected at different time intervals. First, the raw data of the 240 populations are imported into MATLAB, shuffled, and regrouped for preprocessing. The 240 classification codes, which serve as outputs, are converted from one-dimensional output signals to four-dimensional output signals, and programmed in accordance with “0001” representing normal signals, “0010” representing lost signals, “0100” representing signals biased toward high values, and “1000” representing signals biased toward low values. The 240 populations are then sorted based on a certain rule. 160 populations are randomly addressed as training samples, while the remaining 80 populations are used as test samples. Both datasets are normalized to complete data preprocessing.

According to the data samples and data preprocessing, the main process of the multi-source data recognition, classification, and prediction fusion algorithm based on the GA-BP model is as described in section 5.

## 5 Experimental and comparative analysis

### 5.1 Data source

**Figure 5:**
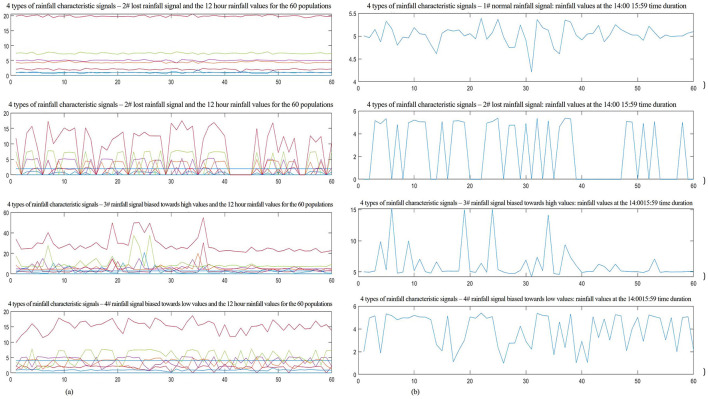
Characteristics of normal and faulty signals and the local enlarged graph. **(A)** Characteristics signals of the 60 populations across the 4 types of signals. **(B)** Partially enlarged signals of the 4 types of signal at the 14:0015:59 time duration.

This study is based on meteorological stations located at the same longitude, latitude, and altitude in a specific area of Qinghai Province. Four sensor arrays are deployed at these stations, with each array consisting of several photoelectric sensors used to collect rainfall data. Rainfall data of 19.8291 mm were collected over a 24-h period during 12 time intervals in a selected month during 2022, starting from 20:00 and ending at 20:00 the following day. The data from sensor #1, which recorded normal signals, are used as an example. See Table 1 for details. Data from 240 nodes were selected for training and network testing (240 samples were selected from the collection database, with each sample containing 14 characteristic signals; specifically, 60 normal signal nodes, 60 lost signal nodes, 60 signal nodes biased toward high values, and 60 signal nodes biased toward low values were selected), as shown in Figure 5A. To research and analyze the rainfall characteristics more clearly, the rainfall values of the four signal types from 14:00 to 15:59 are presented separately. In Figure 5B-1, the values fluctuate ~5 mm, a negligible. This can be identified as locally normal signals. The second row shows clear discontinuities in the waveform, suggesting lost data. In the third row, several points exceed 5 mm, indicating signals biased toward high values. In the fourth row, multiple points are below 5 mm and fluctuate significantly, which can be identified as signals biased toward low values.

**Table 1:** Measurements and error value of the raindrop generator.

**Rainfall collection sensor node number**	**Time interval number**	**Time interval**	**Rainfall (mm)**	**Total rainfall in 24 h (mm)**
1	1	20:00–21:59	0	19.8291
	2	22:00–23:59	0	
	3	00:00–01:59	0	
	4	02:00–03:59	0	
	5	04:00–05:59	0	
	6	06:00–07:59	2.0683	
	7	08:00–09:59	0.9218	
	8	10:00–11:59	4.3615	
	9	12:00–13:59	0	
	10	14:00–15:59	5.0135	
	11	16:00–17:59	7.4640	
	12	18:00–19:59	0	

### 5.2 Parameter optimization of GA-BP model

**Figure 6:**
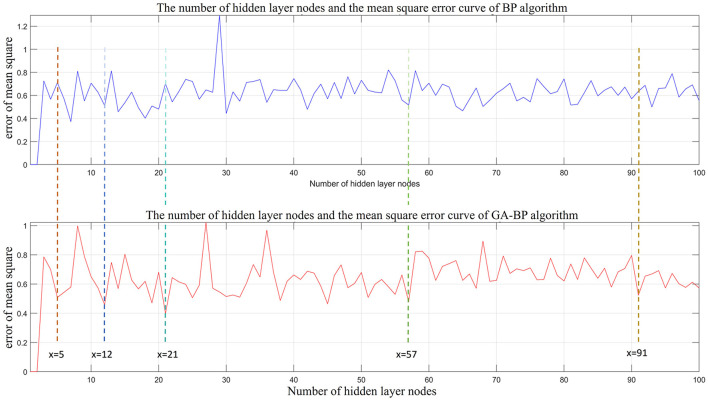
Optimization graph of the hidden layer nodes of BP and GA-BP.

The number of hidden layers is crucial to the recognition and prediction accuracy of the GA-BP network. If the number of hidden layer nodes is too small, the network learning ability is poor, which affects functionality of the network. Alternatively, too many hidden layer nodes can lead to “overfitting”. Numerous studies suggest using the empirical (Equations 13–15) to determine the number of hidden nodes, where *l* is the number of hidden layer nodes; n is the number of input layer nodes; m is the number of output layer nodes; and α is a constant between 0 and 10. In this study, mathematical models of the hidden layers and the BP and GA-BP recognition algorithms were established. Their relationships after 100 iterations in MATLAB are shown in Figure 6. After a comparative analysis of the two graphs, we found that when the number of hidden layer nodes is set to 5, 12, 21, 57, and 91, there are local minimums in the recognition error for the BP and GA-BP algorithms. The specific parameters are shown in Table 2. After considering the recognition errors, simulation time, and comparative analysis of both algorithms, we established that the optimal number of hidden layer nodes is 21.


(13) $$\begin{array}{l} l<n-1 \end{array}$$

(14) $$\begin{array}{l} l<\sqrt{\left(m+n\right)}+\alpha \end{array}$$

(15) $$\begin{array}{l} l\approx \log_{2}n \end{array}$$

**Table 2:** Recognition error values of BP and GA-BP algorithms as well as the modeling and simulation time.

**Algorithm type**	**Number of hidden layer nodes**	**5**	**12**	**21**	**57**	**91**
**BP algorithm**	BP recognition error (mm)	0.71047	0.5105	0.602083	0.516667	0.635417
	BP modeling and simulation time (s)	1.75	2	1.906	4	4.2813
**GA-BP algorithm**	GA-BP recognition error (mm)	0.508333	0.58333	0.397917	0.479167	0.522917
	GA-BP modeling and simulation time (s)	0.625	0.825	0.6825	1.8281	3.0781

### 5.3 Analysis of GA-BP recognition and classification results

**Figure 7:**
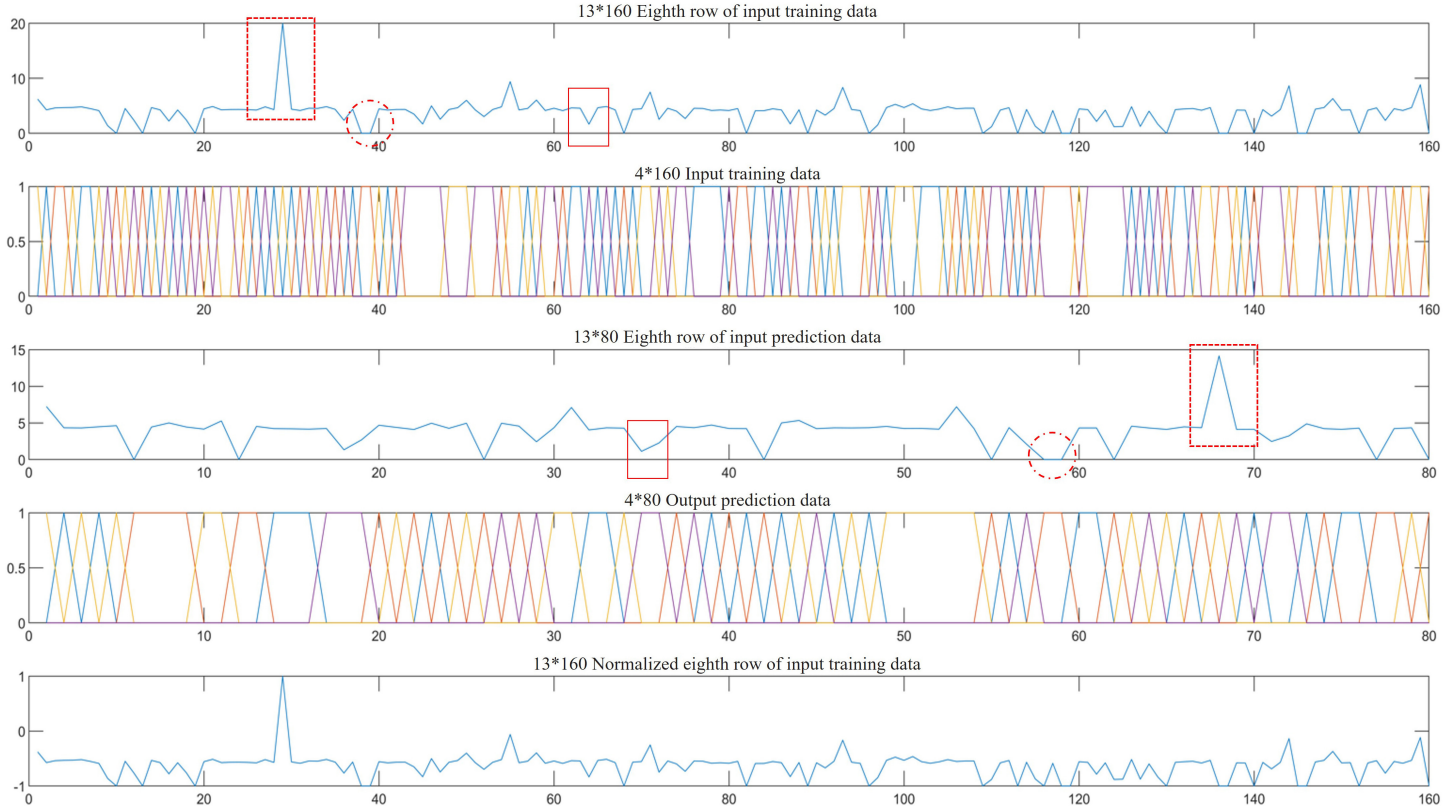
Some input and predicted data, normalized data, and the corresponding output data.

Due to the influence of environmental factors, the data of individual nodes of the sensor array may be too large, too small, and data loss. The 240 original signals were shuffled and regrouped. A total of 160 populations were used as the input training signals for the GA-BP model. Figure 7-1 shows the eighth characteristic signal from the 160 populations, i.e., the rainfall data collected between the 10:00 and 11:59 period (4.3615 mm). Excluding most normal signals, the dashed red box indicates that the data collected by the sensor are biased toward a high value, while the solid red box indicates that the data collected by the sensor are biased toward a low value. The dashed red circle indicates lost data. Faulty sensor nodes similar to the training data are also present in the 80 test populations in the third row of Figure 7. The second and fourth rows of Figure 7 show the training and test output data, respectively. The fifth row mirrors the trend of the first row, which is the result of normalizing the data in the first row. Data normalization helps avoid data disorder caused by inconsistencies in the criteria of different characteristic signals (Kong and Yu, 2022).

**Figure 8:**
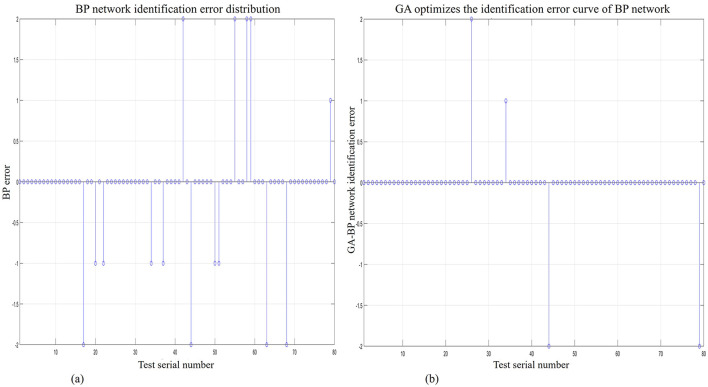
GA-BP network and BP network recognition error discrete graph. **(A)** BP network recognition error discrete graph. **(B)** GA-BP network recognition error discrete graph.

Upon completion of data processing, the BP algorithm model and GA-BP algorithm model were used for recognition and classification. Figure 8A shows the recognition and classification error of the BP neural network algorithm model, while Figure 8B shows the recognition and classification error of the GA-BP algorithm model. After comparing and analyzing the two figures, we found that the GA-BP algorithm model can clearly recognize and classify data with greater accuracy.

**Figure 9:**
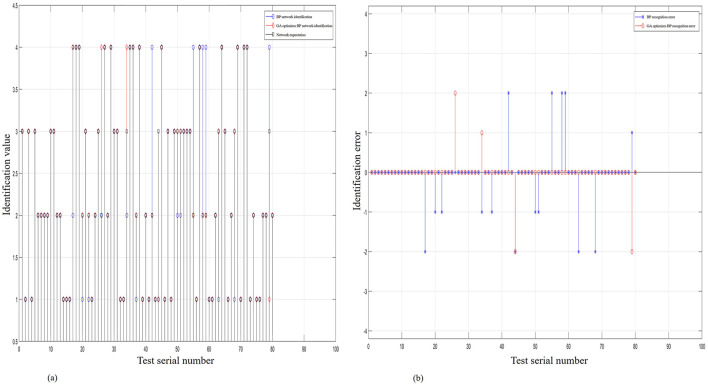
Contrastive chart of the output and error output of GA-BP, BP, and the expected value of rainfall. **(A)** The contrastive chart of the expected output of rainfall of the two models. **(B)** The contrastive chart of error recognition of the two algorithm models.

A comparative analysis was conducted between the expected outputs of the actual rainfall values collected by the 80-sensor nodes, the recognition output values of the BP algorithm model, and the recognition output values of the GA-BP algorithm model. Figure 9A further verifies that the output values of the GA-BP algorithm model are closer to the expected output values. In Figure 9B, the recognition error of the GA-BP model is significantly lower than that of the BP neural network model, indicating that the GA-BP model is more feasible for use in network training and has generalized applicability as a recognition model. The model was run and debugged in MATLAB, the simulation results are shown in Table 3. Compared to the BP algorithm model, the simulation time for the GA-BP algorithm model was reduced from 1.75 to 1.1563 s, indicating improved efficiency. In terms of recognition accuracy, it improved from 69.56% to 95.65% for lost signals; from 68.18% to 86.36% for signals biased toward high values; from 92.85% to 100% for signals biased toward low values; and reached 100% for normal signals. The mean squared error reduced from 0.4875 mm to 0.1625 mm, indicating that the prediction accuracy of the GA-BP algorithm was improved. In summary, the GA-optimized BP neural network (GA-BP algorithm model) outperforms the single BP neural network in fault recognition and classification and is also more efficient with higher prediction accuracy.

**Table 3:** Contrastive analysis of the recognition accuracy and mean square error of GA-BP and BP.

Simulation time for each algorithm (s)	BP model	1.75			
	GA-BP model	1.1563			
Recognition accuracy (%)	BP model	100	69.56522	68.18182	92.85714
	GA-BP model	100	95.65217	86.36364	100
Mean squared error (mm)	BP model	0.4875
	GA-BP model	0.1625

### 5.4 Analysis of the prediction fusion results of the GA-BP model

The GA-BP algorithm model was used for fault recognition and classification, with the located faulty node data input into the GA-BP-based fitting and prediction model to achieve fusion output. Based on the data collected from 80 nodes and input into the GA-BP recognition algorithm model, the faulty nodes were located and classified, as shown in Table 4.

**Table 4:** Fault type and fault location node.

**Fault type**	**Located node**
Signal biased toward high values (7)	6, 8, 22, 29, 33, 57, 68
Signal biased toward low values (5)	3, 39, 45, 52, 73
Lost signal (8)	7, 11, 23, 31, 41, 42, 55, 69

**Figure 10:**
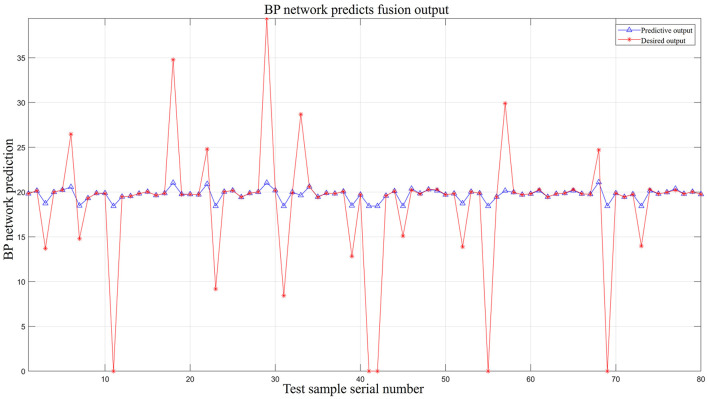
BP network predicts fusion output.

In Figure 10, the red asterisk line represents the expected output values, i.e., the total sum of the rainfall values collected by the 80 sensors over the last 12-h interval. The normal signal output value is approximately 19.8291 mm. As shown in Table 4, there are 20 faulty nodes in the 80-sensor array, as a result of which the red star line fluctuates considerably, indicating data anomalies at these nodes. The blue triangle solid line represents the output values after the data are processed by the BP neural network model. The output curve is clearly smooth and is close to the normal output signal value of 19.8291 mm, therefore achieving the goal of data fusion output.

**Figure 11:**
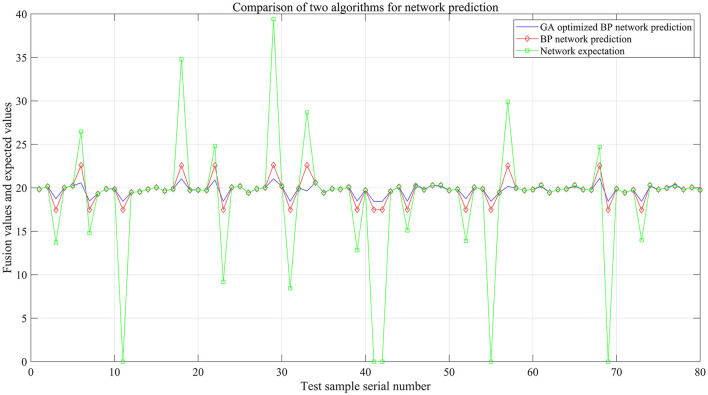
Contrastive analysis diagram of the fusion output of GA-BP, BP, and the expected value of rainfall.

After the data are processed by the GA-BP algorithm model, the blue solid line in Figure 11 represents the output curve of the GA-BP model. This curve is smoother than the other two curves and is closer to the standard daily rainfall value. A comparison of the prediction results shows that in terms of faulty node troubleshooting, fitting, and fusion output, GA-BP > BP > “expected output.”

**Figure 12:**
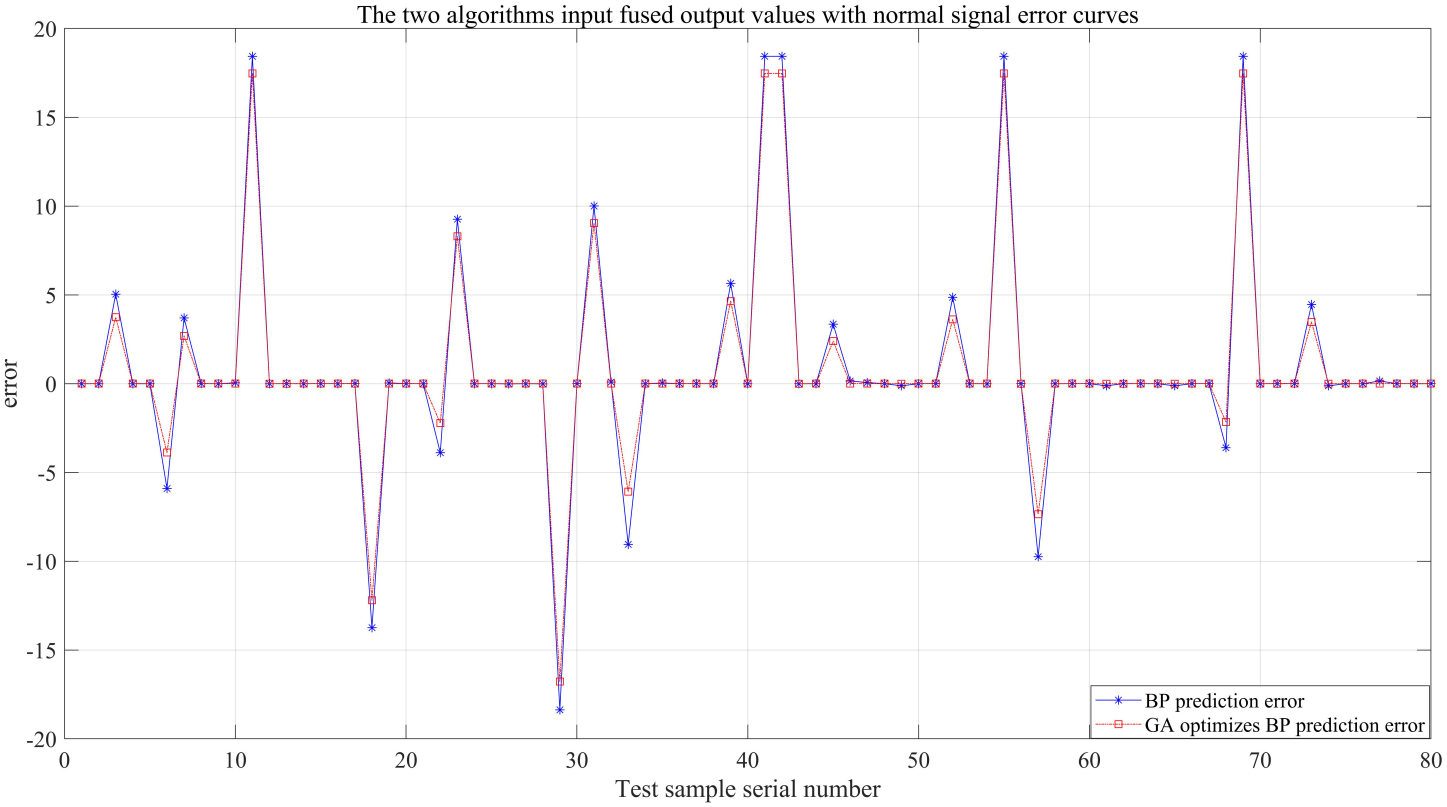
Contrastive diagram of the error output of the two algorithms.

To more intuitively compare the GA-BP algorithm model and the BP algorithm model in terms of prediction fusion effects, the fusion output values of both models were compared with the actual rainfall values for the day, as shown in Figure 12. The experimental simulation results indicate that the fusion output of the GA-BP algorithm model is closer to the actual values (see Table 5 for details), and the simulation time decreased from 2.5781 s to 0.20313 s. The mean squared error also decreased from 33.7986 mm to 30.3027 mm.

**Table 5:** Fusion output values of the two algorithm models.

**Fault type**	**Faulty node**	**Actual value (mm)**	**Fusion value predicted by the BP algorithm model (mm)**	**Fusion value predicted by the two-layer GA-BP algorithm model (mm)**	**Expected value (mm)**
Lost signal	7 (signal lost during a partial time interval)	14.7852	18.4816	19.4730	19.8291
	11	0	18.4333	19.3237	
	23 (signal lost during a partial time interval)	9.1770	18.4373	19.4733	
	31 (signal lost during a partial time interval)	8.4280	18.4331	19.4731	
	41	0	18.4362	19.4724	
	42	0	18.4175	19.4930	
	55	0	18.4321	19.4233	
	69	0	18.4337	19.3471	
Signal biased toward high values	6	26.4699	20.5686	19.5886	
	18	34.7784	21.0361	20.5771	
	22	24.7940	20.9024	19.5687	
	29	39.4031	21.0368	20.6240	
	33	28.6831	19.6337	20.5965	
	57	29.8991	20.1493	19.5390	
	68	24.7061	21.1020	20.5439	
Signal biased toward low values	3	13.7031	18.7371	19.4419	
	39	12.8361	18.4821	19.4816	
	45	15.0951	18.4393	19.4868	
	52	13.8791	18.7350	20.4928	
	73	13.9741	18.4296	19.4462	

## 6 Conclusion

This study discusses the random and diverse sensor faults that occur during multi-source data fusion, which lead to low accuracy of sensor fault characteristic recognition and classification, and inefficient handling of complex issues by algorithms. In addition, after faults are recognized and located, the processing of data from the faulty nodes and fusion output results are not ideal. This study proposes a multi-source data recognition, classification, and prediction fusion algorithm based on a GA-BP model. First, the data of the 240 populations collected by the sensor arrays were preprocessed through normalization and then divided into training and test datasets to construct an appropriate training network. Second, an appropriate GA and BP neural network model was built to establish a GA-BP algorithm model for multi-source data fault recognition and classification. Finally, an appropriate GA-BP network structure was created, with the recognized fault data serving as the input to the second-layer GA-BP algorithm model in order to achieve data fitting and fusion output.

The experimental simulation results show that:

- The GA-BP algorithm model all outperforms a single BP neural network in terms of operational efficiency. At the fault recognition and classification stage, the GA-BP model reduced the run time by 0.6 s compared to the BP model. As the size of the sensor array increased, the simulation time disparity increased. At the data fusion stage, the program ran more efficiently, and the GA-BP model reduced the run time by 2.37497 s compared to the BP neural network model.
- Compared to the BP neural network model, the GA-BP model improved the recognition accuracy for lost signals from 69.56% to 95.65% and improved the accuracy for signals biased toward high values from 68.18% to 86.36%, the accuracy for signals biased toward low values from 92.85% to 100%, and the accuracy for normal signals to 100%, indicating that normal signals were all correctly identified. This further validates the superior recognition and fusion accuracy of the GA-BP model.
- On the basis of the existing BP neural network, the fault recognition output of the first layer served as the input to the second-layer GA-BP algorithm model. An appropriate BP network structure was selected and various GA parameters were constructed, with specific sub-functions written for fault recognition and troubleshooting. The mean squared error decreased from 33.7986 mm to 30.3027 mm, and the output data were smooth with less fluctuation. This enhanced the robustness and generalization ability of the system.

This thesis has analyzed the rainfall data but has not yet carried out research on larger and more complex datasets and more meta-heuristic optimization methods, which is one of the directions for future research and exploration.

## Data Availability

The datasets presented in this article are not readily available because the practical data of the paper may be applied to further research. Requests to access the datasets should be directed to xiongzhuang0919@163.com.
